# Supplementary figures and images for: Biases in Routine Influenza Surveillance Indicators Used to Monitor Infection Incidence and Recommendations for Improvement
Source: Influenza Other Respir Viruses. 2024 Dec 1;18(12):e70050. doi: 10.1111/irv.70050 (PMC11608885; doi:10.1111/irv.70050)

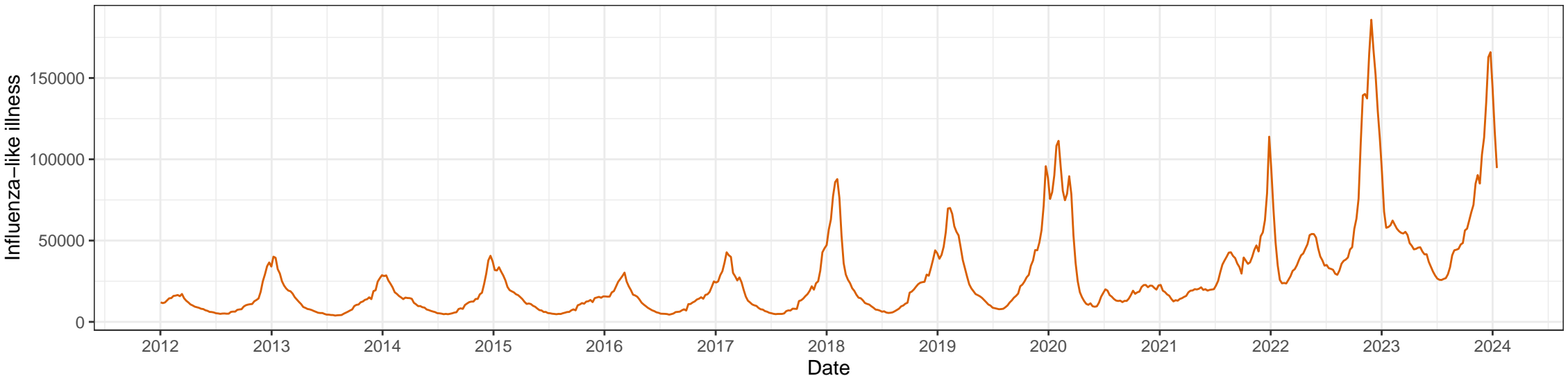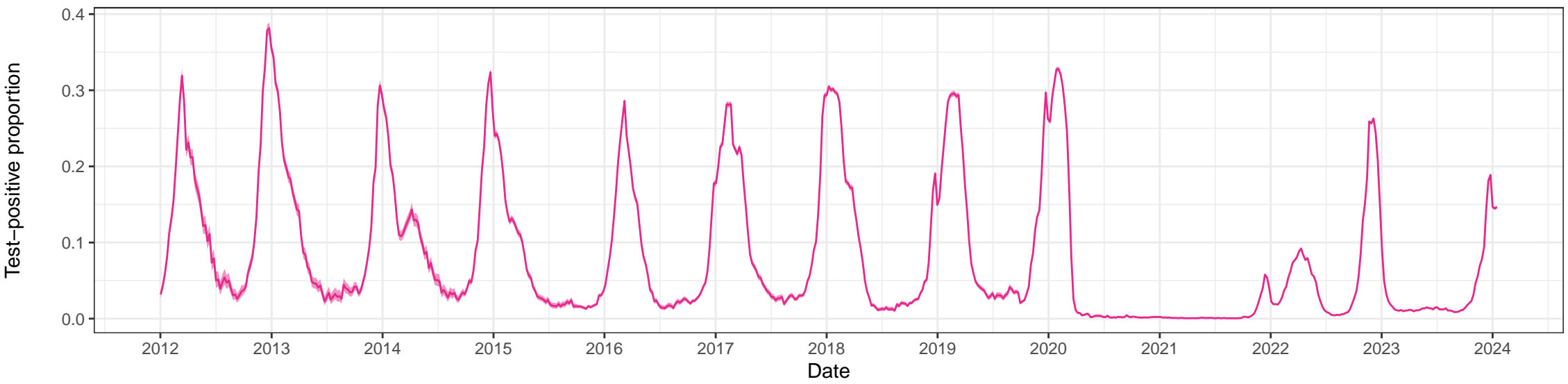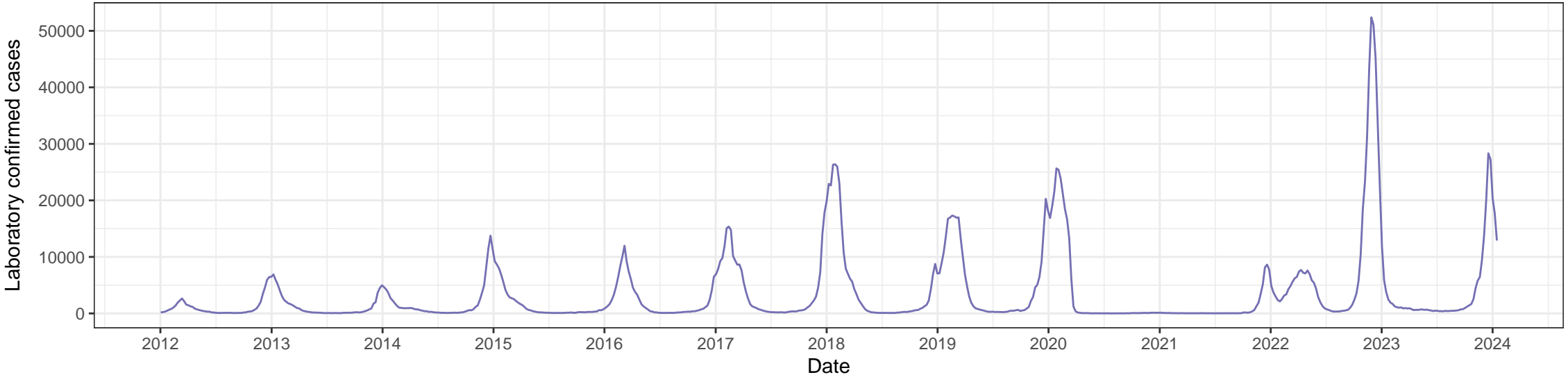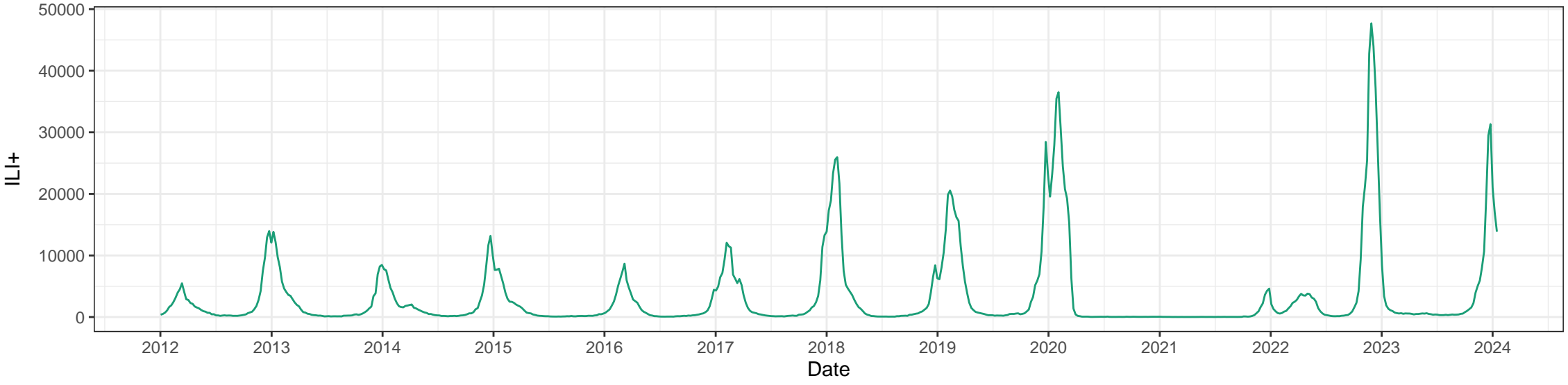

Supplement: Supplementary file 1 — SFig1.pdf [file IRV-18-e70050-s002.pdf]

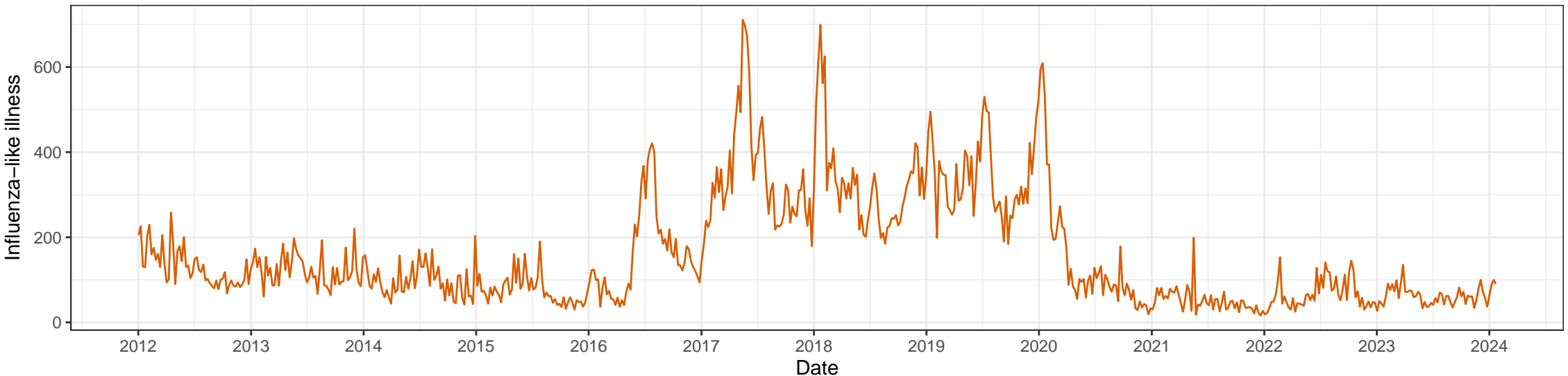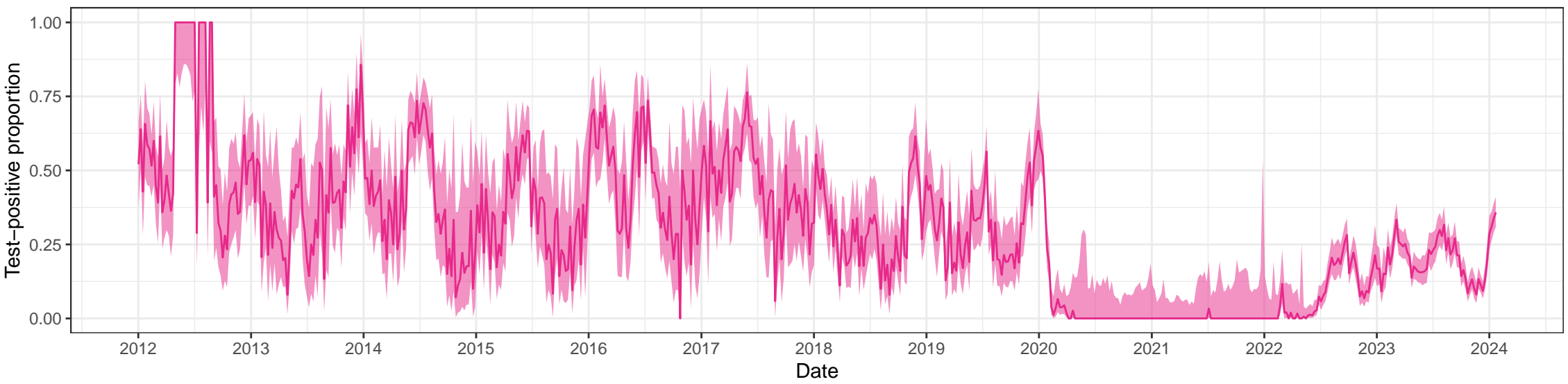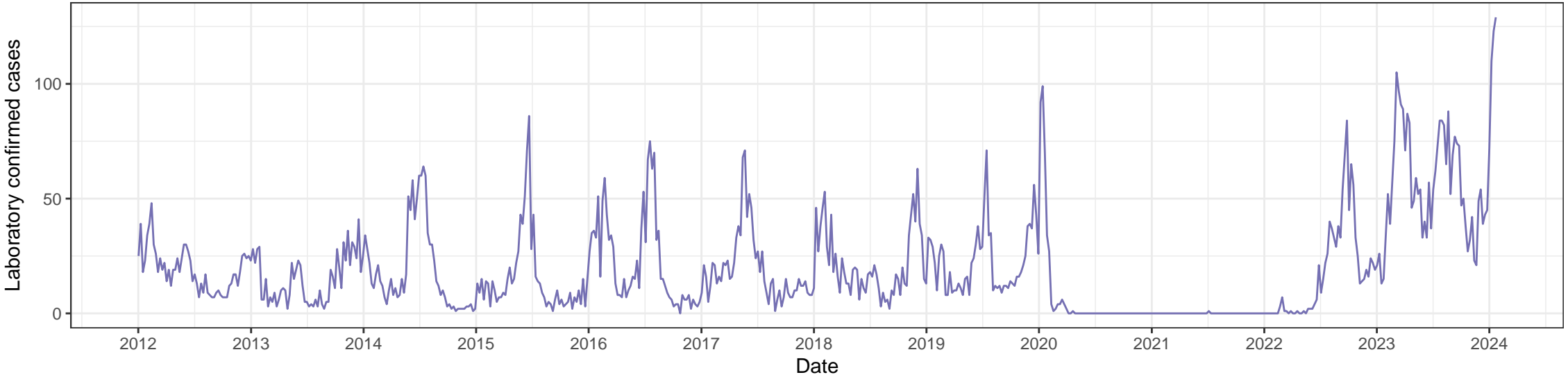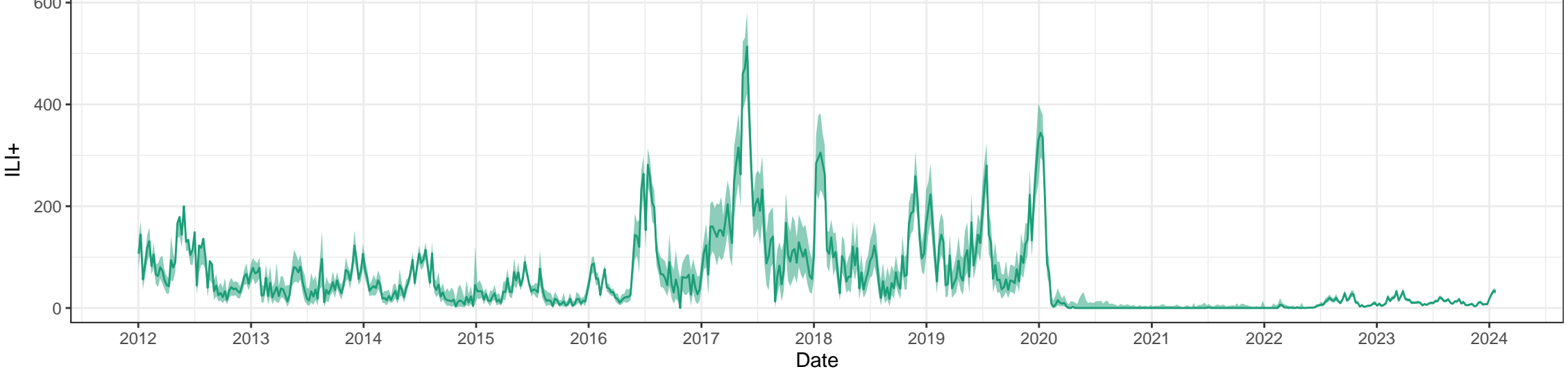

Supplement: Supplementary file 2 — SFig2.pdf [file IRV-18-e70050-s003.pdf]

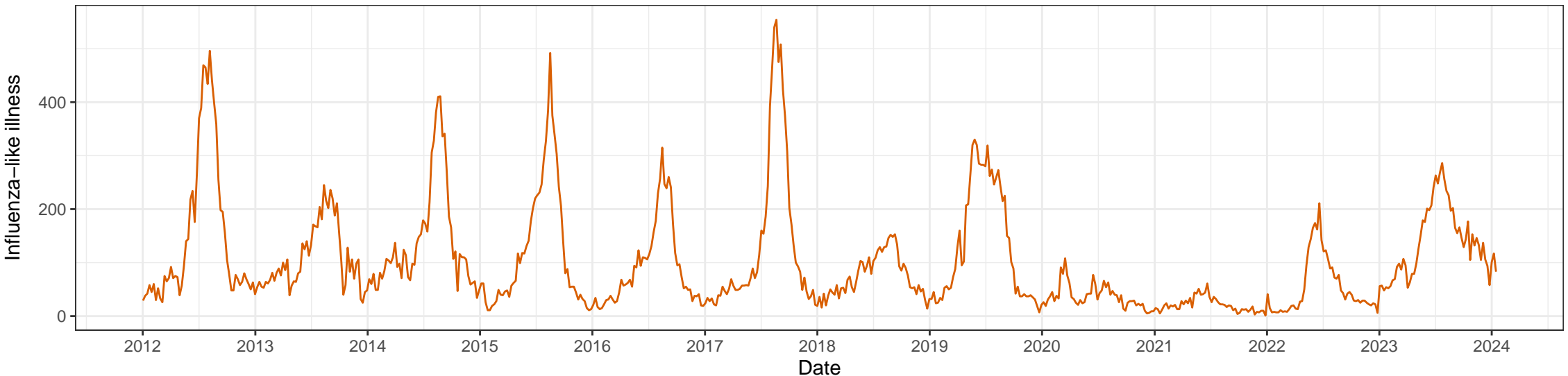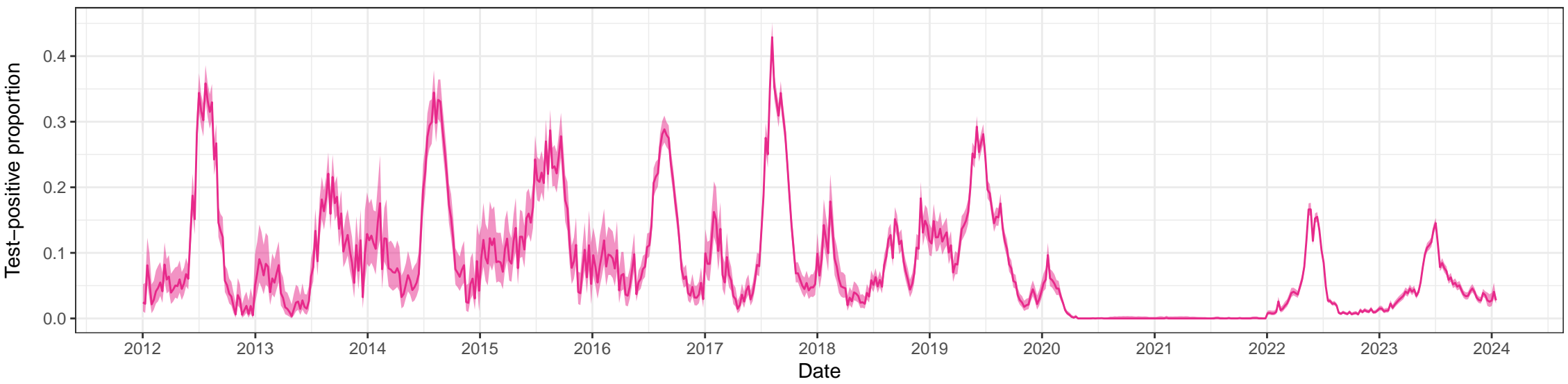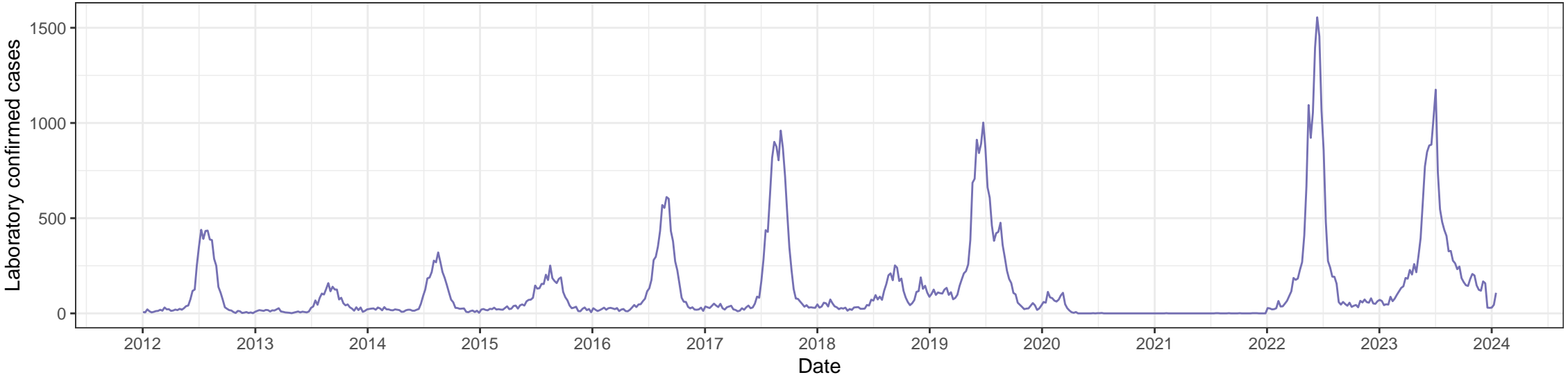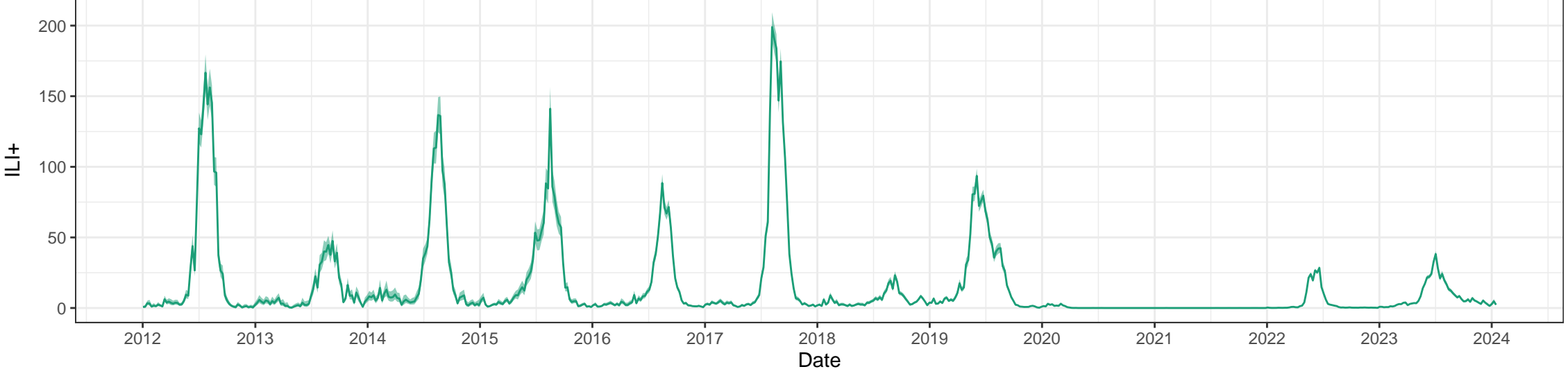

Supplement: Supplementary file 3 — SFig3.pdf [file IRV-18-e70050-s001.pdf]

**A**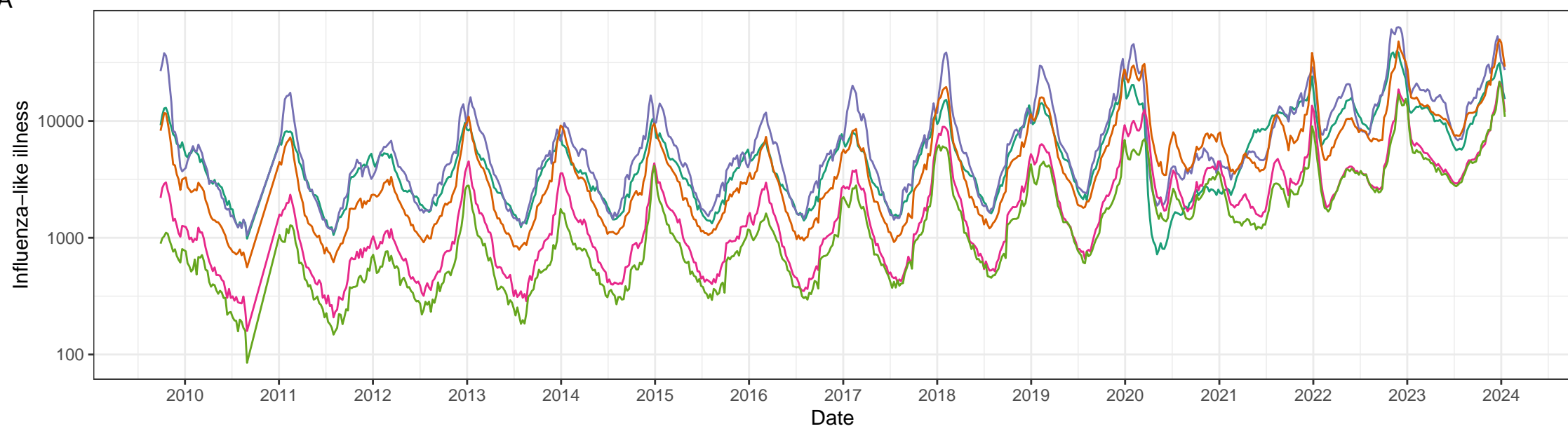**B**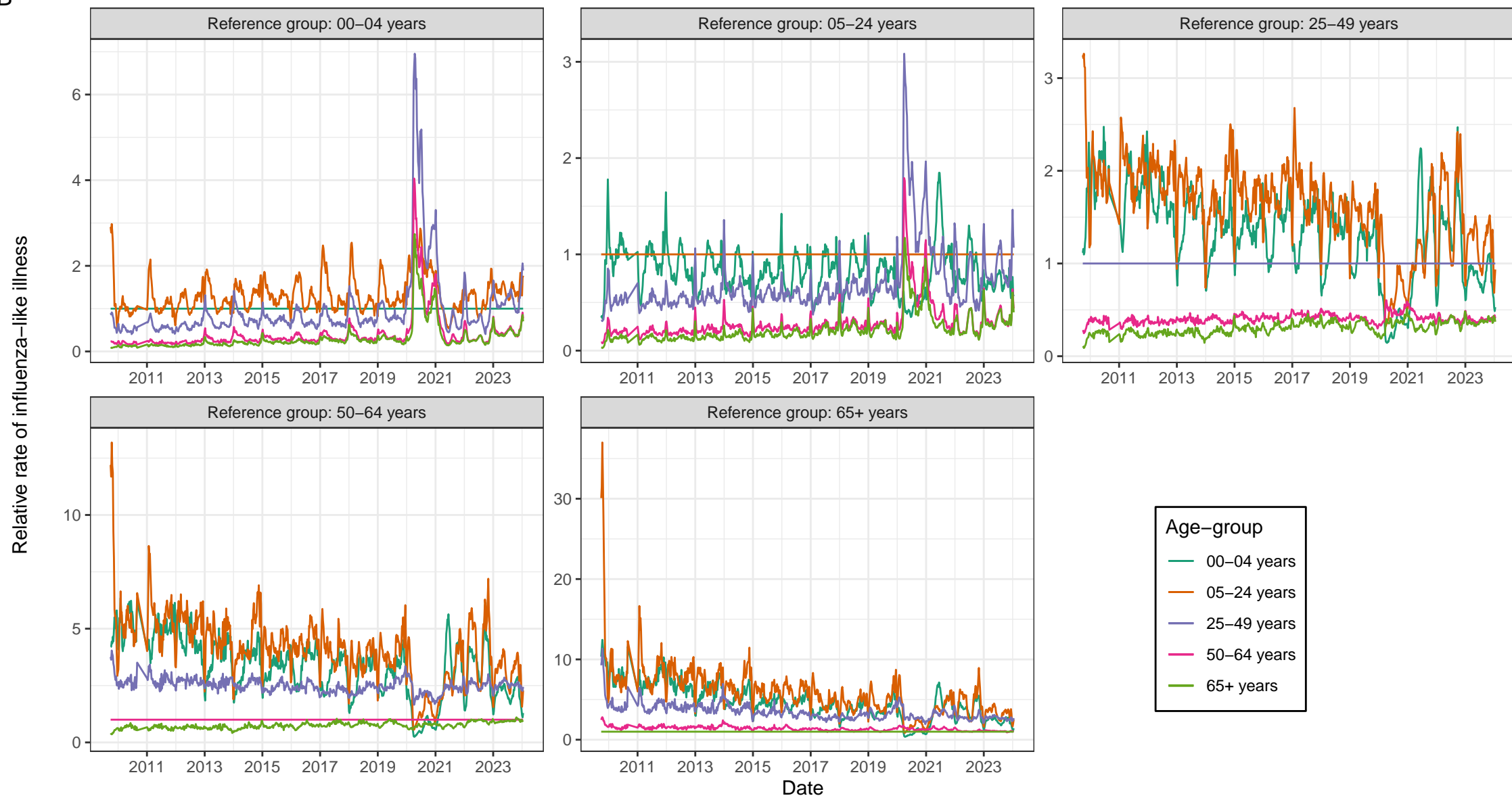

Supplement: Supplementary file 5 — SFig5.pdf [file IRV-18-e70050-s005.pdf]
