# Supplementary material for: Biases in Routine Influenza Surveillance Indicators Used to Monitor Infection Incidence and Recommendations for Improvement
Source: Influenza Other Respir Viruses. 2024 Dec 1;18(12):e70050. doi: 10.1111/irv.70050 (PMC11608885; doi:10.1111/irv.70050)
Supplement: Supplementary file 4 — SFig4.pdf [file IRV-18-e70050-s004.pdf]

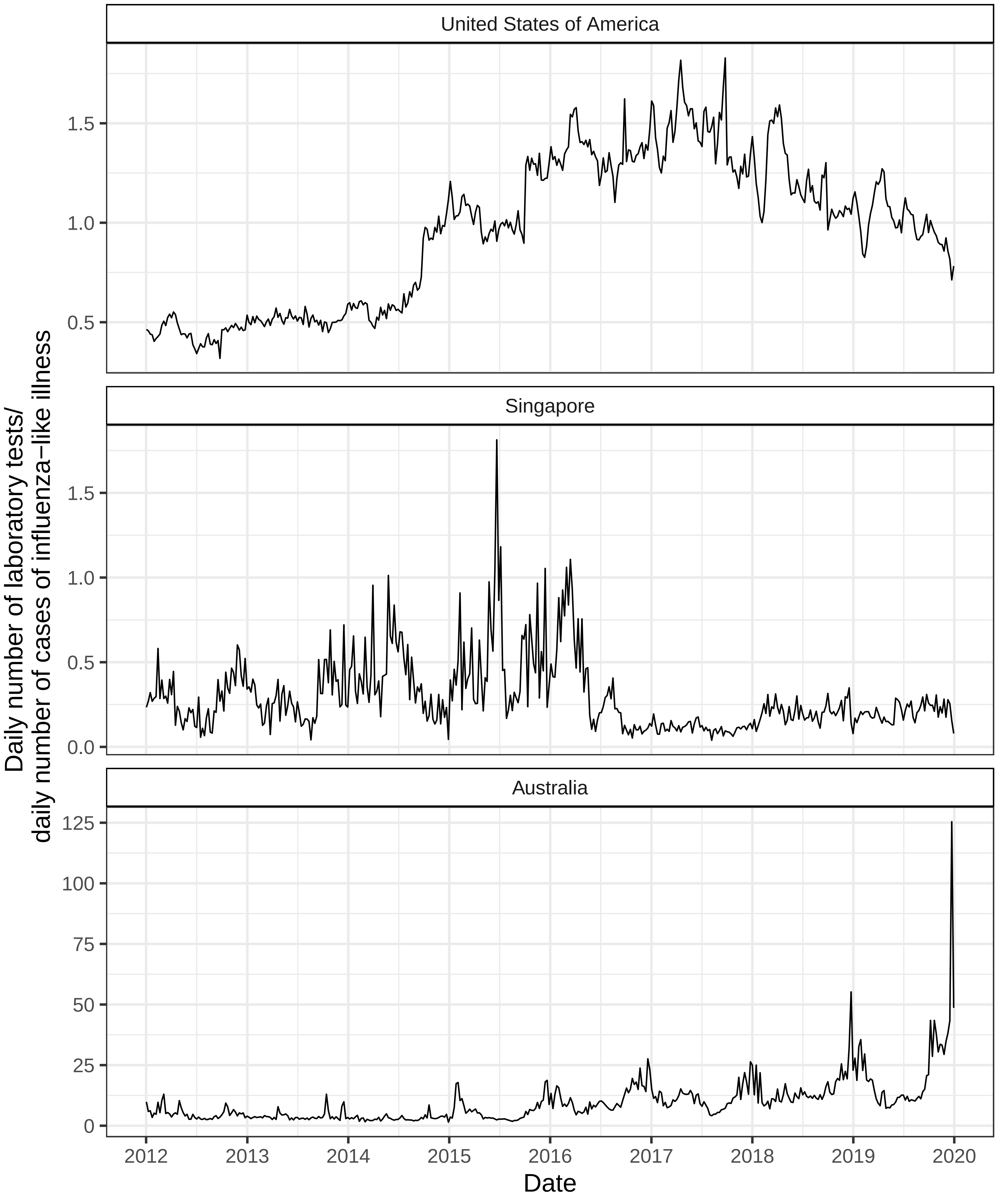
United States of America
Singapore
Australia
Daily number of laboratory tests/
daily number of cases of influenza−like illness
Date
0.5
1.0
1.5
0.0
0
25
50
75
100
125
2012
2013
2014
2015
2016
2017
2018
2019
2020
